# An LNA gapmer antisense oligonucleotide inhibitor of human NNMT

**DOI:** 10.1016/j.omtn.2025.102761

**Published:** 2025-11-13

**Authors:** Suxiang Chen, Bal Hari Poudel, Rakesh Naduvile Veedu

**Affiliations:** 1 Personalised Medicine Centre, Health Futures Institute, Murdoch University, Murdoch, WA 6150, Australia; 2 Precision Nucleic Acid Therapeutics, Perron Institute for Neurological and Translational Science, Nedlands, WA 6009, Australia; 3 ProGenis Pharmaceuticals Pty Ltd., Bentley, WA 6102, Australia; 4 SynGenis Pty Ltd., Bentley, WA 6102, Australia

## Main text

Nicotinamide N-methyltransferase (NNMT), an enzyme that catalyzes the methylation of nicotinamide (a form of vitamin B3) using S-adenosyl-L-methionine (SAM) as the methyl donor to produce 1-methyl-nicotinamide and S-adenosyl-L-homocysteine (SAH), has been reported to be implicated in the pathogenesis of different disorders including metabolic syndrome,^1^ inflammatory autoimmune disease,^2^ age-related conditions,^3^ and cancers.^4^ As an intracellular protein, NNMT does not lend itself to antibody-based targeted therapy, therefore, RNA-targeting antisense oligonucleotides (ASOs) are considered a feasible option for inhibitor development. Although a 5-10-5 2′-*O*-methoxyethyl (2′-MOE) gapmer ASO with a full phosphorothioate (PS) backbone has been developed as NNMT inhibitor against diet-induced obesity more than a decade ago,^5^ this ASO specifically targets the *Nnmt* mRNA of mouse instead of human. Thus, it only serves as a research tool but not a potential drug. Recently, Tomoaki Hara et al.^6^ reported their identification of a 3-12-3 locked nucleic acid (LNA)-PS gapmer ASO named hNNMT-897-LNA(18) targeting the human *NNMT* mRNA after *in silico* and *in vitro* screening. Their work not only provides an efficient ASO-based human NNMT inhibitor exhibiting antitumor activity, more importantly, but also paves the way for future studies on repurposing this lead ASO compound as potential therapeutics for other NNMT-related disorders.

ASOs have been widely used to inhibit gene expression for both research and therapeutic purposes. Although steric-blocking ASOs (usually fully composed of sugar-modified RNA-like nucleotide analogues) can downregulate gene expression through different mechanisms (e.g., translational repression or exon skipping mediated reading frameshift), RNase H-dependent ASOs (full DNA or gapmer-like ASO containing a central DNA gap flanked by sugar-modified wing sequences) are far more commonly used for gene knockdown purpose. As of October 2025, eight RNase H-dependent ASOs have been approved by the U.S. Food and Drug Administration (FDA) and/or European Medicines Agency (EMA) for clinical use. Among them, except for fomivirsen, a 21-mer DNA-based ASO drug with a full PS backbone, the rest of the approved RNase H-dependent ASOs are all 20-mer 5-10-5 2′-MOE gapmers with a full PS backbone (mipomersen, inotersen, volanesorsen, and olezarsen) or mixed phosphodiester (PO)–PS linkages (tofersen, eplontersen, and donidalorsen).

In addition to 2′-*O*-methyl (2′-OMe) and 2′-MOE, in many studies, LNA also has been applied as the chemistry of wing sequences in gapmer-like ASOs due to enhanced RNA binding affinity and improved potency for reducing target mRNA. However, LNA-modified ASOs could cause hepatotoxicity through binding to liver protein^7^ or inducing off-target RNA knockdown.^8^ Exploration of novel nucleotide analogues as building blocks of gapmer wings has been ongoing. For instance, 2′,4′-constrained 2′-*O*-ethyl (cEt, an analogue of LNA), 2′,4′-constrained 2′-*O*-methoxyethyl (cMOE, an analogue of both 2′-MOE and LNA), and even phosphorodiamidate morpholino oligomer (PMO) have been reported to construct the wing sequences of gapmer ASOs.^9^

**Figure 1:**
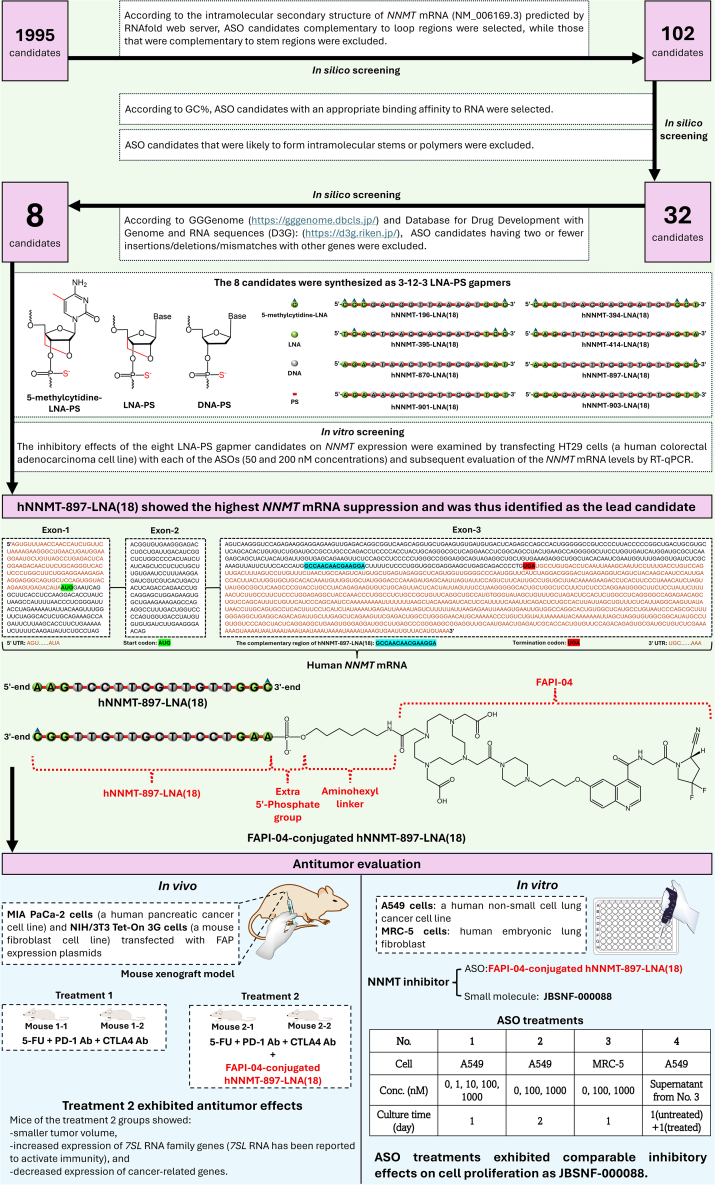
Identification of lead ASO candidate as NNMT inhibitor and antitumoral evaluation of this ASO

In this work, an LNA gapmer ASO (with a full PS backbone) complementary to a stretch of 18-nt in exon-3 of human *NNMT* mRNA was identified as a lead inhibitor by both *in silico* and *in vitro* screens (Figure 1). Initially, 1995 antisense sequences were designed and narrowed down to 102 hit sequences based on the intramolecular secondary structure of human *NNMT* mRNA predicted by RNAfold web server. Specifically, ASO candidates complementary to stem regions were excluded, while those that were complementary to loop regions were retained. This is because ASOs designed in stem regions need to undergo strand invasion of RNA-RNA duplex to form ASO-RNA duplex, resulting in reduced accessibility to mRNA for ASOs designed in stem regions compared to those designed in loop regions. Subsequent *in silico* screens were performed by retaining ASO candidates with an appropriate RNA binding affinity according to GC-content and removing those that were likely to form intramolecular stems or polymers; as a result, the number of hit sequences was further reduced to 32. A final *in silico* screen was performed by excluding ASOs having only one or two mutations (mismatches/insertions/deletions) with other genes to avoid the risk of causing hybridization-dependent unintended toxicity (off-target effects). Although it is known that two mismatches in a 20-mer ASO sequence are usually sufficient to avoid effects on non-target RNAs, it has been reported that LNA modifications could induce off-target effects by increasing ASOs’ tolerance of mismatches^10^; therefore, in this work, ASO candidates possessing at least three mutations with non-*NNMT* genes were retained. As a result, the number of hit ASO sequences was narrowed down to 8 at the end of the multiple *in silico* screens (Figure 1). These sequences were then synthesized as 3-12-3 LNA gapmers with full PS linkages, which was followed by an *in vitro* screening of inhibitory effect of these gapmers on human *NNMT* mRNA in HT29 cells (a human colorectal adenocarcinoma cell line). As a result, hNNMT-897-LNA(18) was identified as the best-performing candidate inhibitor of NNMT.

Next, as NNMT is highly expressed in the stroma of cancer tissues, hNNMT-897-LNA(18) was conjugated with FAPI-04 (Figure 1), a fibroblast activation protein (FAP, a cell surface protein specifically expressed in cancer-associated fibroblasts [CAFs])-binding compound to enable it to be specifically taken up by CAFs, the main constituent of tumor stroma. FAPI-04-conjugated hNNMT-897-LNA(18) was then evaluated for its anticancer effects both *in vivo* and *in vitro*. First, a mouse xenograft model was set up by subcutaneous injection of a mixture of MIA PaCa-2 cells (a human pancreatic cancer cell line) and NIH/3T3 Tet-On 3G cells (a mouse fibroblast cell line) transfected with FAP expression plasmids into NOD/SCID mice (an immunodeficient mouse strain). Two mice were intravenously administrated by 5-fluorouracil (5-FU) and immune checkpoint inhibitors (PD-1 antibody and CTLA4 antibody), while the other two mice were treated with 5-FU, PD-1 antibody, CTLA4 antibody, and FAPI-04-conjugated hNNMT-897-LNA(18). The latter treatment (with ASO) exhibited enhanced antitumor effects compared to the former treatment (without ASO) that the mice from the latter group exhibited smaller tumor volume, increased expression of RN7SL3, RN7SL2, RN7SL767P, RN7SL5P, RN7SL4P RNAs (7SL RNAs can activate immunity), and decreased expression of cancer-related genes (*MYC*, *CCND1*, *SNAI2*, *TGFB2*, and *MLH1*). On the other hand, *in vitro* investigation of anticancer activity of FAPI-04-conjugated hNNMT-897-LNA(18) on A549 cells (a human non-small cell lung cancer cell line) was also performed. At 1000 nM concentration, FAPI-04-conjugated hNNMT-897-LNA(18) showed significant inhibition on the proliferation of A549 cells after 1 day of treatment and achieved comparable inhibition as JBSNF-000088 (a small molecule NNMT inhibitor) after 2 days of treatment. FAPI-04-conjugated hNNMT-897-LNA(18) and JBSNF-000088 were also used to treat MRC-5 cells (human embryonic lung fibroblast) separately for 1 day, the supernatants of cell culture were then collected and used to treat A549 cells. Again, at 1000 nM concentration, FAPI-04-conjugated hNNMT-897-LNA(18) displayed comparable inhibition on A549 cell viability as JBSNF-000088.

In summary, this is the first report that an LNA gapmer ASO-based inhibitor of human NNMT, i.e., hNNMT-897-LNA(18) has been developed.^6^ Subsequent studies of FAPI-04-conjugated hNNMT-897-LNA(18) showed antitumor effects *in vivo* and *in vitro*. Their work definitely lays the foundation for further development of ASO-based NNMT inhibitor as potential therapeutics for NNMT-related diseases, in particular, cancers. However, we suggest that a scrambled ASO control of hNNMT-897-LNA(18) should be included in all future *in vitro* and *in vivo* studies. In addition, the toxicity of the ASO on normal human cells (other than cancer cells) needs to be further evaluated and improved, since hNNMT-897-LNA(18) also showed inhibitory effect on MRC-5 cells as JBSNF-000088 did. Finally, we believe that interested researchers may further optimize the chemistry of the ASO, for instance, incorporation of PMO as the wings of gapmer, control of stereochemistry,^9^ or adoption of a chimeric PO–PS backbone, prior to performing disease-specific assays.

## Declaration of interests

The authors declare no competing interests.
